# DAMP-Mediated Activation of NLRP3-Inflammasome in Brain Sterile Inflammation: The Fine Line between Healing and Neurodegeneration

**DOI:** 10.3389/fimmu.2014.00099

**Published:** 2014-03-17

**Authors:** Anna Rubartelli

**Affiliations:** ^1^Cell Biology Unit, IRCCS AOU San Martino-IST, Genova, Italy

**Keywords:** stress, neurotoxicity, glia, IL-1beta, NLRP3-inflammasome

The sterile inflammatory response that occurs following acute brain insults such as stroke, hemorrhage, or trauma is detrimental and largely contributes to the incidence of neurodegeneration (1). Thus, like in all organs, inflammation may switch from a protective/homeostatic role to a destructive effect also in the central nervous system (CNS). Inflammation-mediated neurotoxicity occurs as a consequence of glial cell dysregulation and overactivation: while moderate CNS damage evokes protection by microglia, intensive acute activation renders these cells harmful, potentially impairing neuronal activity (2). Although much evidence indicates that the IL-1 system is involved in this adverse process, the expression, regulation, and role of NLRP3-inflammasome in the CNS are poorly understood (3). In particular, studies on inflammasome activation by damage associated molecular patterns (DAMPs) released following brain injury have been hindered so far by the failure to induce inflammasome activation under completely sterile conditions, that is, without a priming stimulus by pathogen products (pathogen associated molecular patterns, PAMPs).

In their interesting article, Savage and coworkers (4) show that DAMPs such as ATP, monosodium urate, and calcium pyrophosphate dehydrate crystals induce the production of IL-6, CXCL1, and proteases by glial cells but have no effect on the expression of IL-1α or IL-1β. Remarkably, they also show that the acute phase protein serum amyloid A (SAA) acts as a priming stimulus driving levels of IL-1β expression comparable to those induced by lipopolysaccharide. Therefore, an endogenous stress protein such as SAA, which we can define a DAMP-like molecule, primes glial cells, and allows other DAMPs to activate the inflammasome and consequently the secretion of IL-1β. Thus, the first merit of this study is the demonstration that IL-1β secretion may be induced by DAMPs via activation of the NLRP3-inflammasome on glial cells in the absence of an initial priming stimulus by PAMPs, that is, in a condition of purely sterile inflammation.

The excellent study of Savage and coworkers is also important as it stresses that inflammatory responses in the brain may be influenced by both local DAMPs and circulating inflammatory mediators. In fact, although in the absence of injury the brain is protected by the blood brain barrier (BBB), which keeps microglia isolated from potential blood-derived priming stimuli, the acute inflammatory event itself causes the early breakdown of the BBB by glia-produced proteases, allowing the penetration of circulating inflammatory mediators into the brain. These include SAA that is strongly upregulated in blood at the same time as disruption of BBB occurs, and acts as a priming factor for IL-1β production in glial cells.

It is worth stressing that the BBB breakdown not only allows the passage of serum components, but also of circulating cells including monocytes. A series of recent studies [reviewed in Ref. (2)] indicate that blood-derived monocytes in injured brain perform crucial roles that cannot be accomplished by microglia, their resident counterparts. According to this view when glial cells can no longer provide protection, monocytes are recruited to the damaged CNS; there, they work not as microglial replacements but rather as assistant cells that carry out activities that resident glial cells are not (anymore) able to perform. The definition of the different pro- and anti-inflammatory properties of glia and blood-derived macrophages, including the repertoire of produced cytokines, would be of great help for our understanding of the acute inflammatory response in the brain.

An additional relevant finding came from the *in vivo* experiments in IL-1α/β double KO mice, which showed that IL-1 is required for IL-6 and CXCL1 production after cerebral ischemia. Therefore, in acute brain injury the contribution of DAMPs to brain inflammation occurs at various levels: by directly stimulating the production of pro-inflammatory mediators by glial cells, by contributing to BBB injury through induction of release of various proteases, and by triggering IL-1 release from primed cells. Then IL-1 markedly enhances damage-induced inflammatory responses.

The observations reported in this article raise a number of stimulating questions. Some of these concern the mechanism(s) of inflammasome activation in brain. While the key role of K^+^ and Ca^2+^ flux modulations in activating NLRP3-inflammasome in peripheral monocytes/macrophages is widely recognized, whether similar fluctuations are also required to activate inflammasome in glial cells is unclear. The different microenvironment may represent a problem since in the brain Na^+^, K^+^, Ca^2+^ concentrations must be tightly regulated as inward and outward transport of these ions controls neuron excitation. Strong ion fluxes such as those described in activated monocytes/macrophages could be detrimental for neuron survival. Along this line, a previous study reported that microglia, in response to pattern recognition receptor engagement by certain stimuli, enter an overactivated state and release reactive oxygen species (ROS) that cause neurotoxicity (5). In monocyte/macrophages, inflammasome activation requires a redox signaling with both ROS and antioxidant systems implicated in the correct out come of the process (6). Likewise, ROS and the consequent redox signaling may play a role in inflammasome activation also in the brain. Thus, the involvement of ROS produced by overactivated microglia in this process deserves further investigations.

Finally, an obvious extension of these studies will be the search of the conditions that promote cytokine-mediated neuronal degeneration over survival. An important issue is which cytokines of the IL-1 family are produced by glial cells. Among them, IL-18 is of particular interest since like IL-1β is processed by the NLRP3-inflammasome, is induced following physical/emotional stress and triggers microglial activation (7). Also the production of IL-1Ra is highly relevant, since elevation of IL-1Ra is of key importance for quenching the inflammatory response at the IL-1R1 as part of an autoregulatory loop (8). A different level of production of this cytokine by microglia and infiltrating blood-derived monocytes might also explain the different role of these cell populations in acute brain injury. Like in other inflammatory diseases, induction, or pharmacological application of IL-1Ra may be beneficial in neurotrauma and save neurons from the toxic effects of overactivated microglia.
